# Ventilator-Associated Pneumonia, Multidrug-Resistant Bacteremia and Infection Control Interventions in an Intensive Care Unit: Analysis of Six-Year Time-Series Data

**DOI:** 10.3390/antibiotics11081128

**Published:** 2022-08-19

**Authors:** Amalia Papanikolopoulou, Helena C. Maltezou, Athina Stoupis, Anastasia Pangalis, Christos Kouroumpetsis, Genovefa Chronopoulou, Yannis Kalofissoudis, Evangelos Kostares, Fotini Boufidou, Maria Karalexi, Vasiliki Koumaki, Nikos Pantazis, Athanasios Tsakris, Maria Kantzanou

**Affiliations:** 1 Clinical Pharmacology Department, Athens Medical Center, 15125 Athens, Greece; 2 Directorate of Research, Studies and Documentation, National Public Health Organization, 15123 Athens, Greece; 3 Clinical Infectious Diseases Department, Athens Medical Center, 15125 Athens, Greece; 4 Biopathology Department Athens Medical Center, Marousi, 15125 Athens, Greece; 5 Intensive Care Unit, Athens Medical Center, Marousi, 15125 Athens, Greece; 6 Quality Assurance Department, Athens Medical Center, Marousi, 15125 Athens, Greece; 7 Department of Microbiology, School of Medicine, National and Kapodistrian University of Athens, 75 Mikras Asias Street, 11527 Athens, Greece; 8 Neurochemistry and Biological Markers Unit, 1st Department of Neurology, School of Medicine, Eginition Hospital, National and Kapodistrian University of Athens, 11528 Athens, Greece; 9 Department of Hygiene, Epidemiology and Medical Statistics, Faculty of Medicine, School of Health Sciences, National and Kapodistrian University of Athens, 11527 Athens, Greece

**Keywords:** ventilator-associated pneumonia, infection control interventions, time-series analysis, multi-drug resistant pathogens, healthcare-associated infection, hospital

## Abstract

Ventilator-associated pneumonia (VAP) occurs more than 48h after mechanical ventilation and is associated with a high mortality rate. The current hospital-based study aims to investigate the association between VAP rate, incidence of bacteremia from multidrug-resistant (MDR) pathogens, and infection control interventions in a single case mix ICU from 2013 to 2018. **Methods:** The following monthly indices were analyzed: (1) VAP rate; (2) use of hand hygiene disinfectants; (3) isolation rate of patients with MDR bacteria; and (4) incidence of bacteremia/1000 patient-days (total cases, total carbapenem-resistant cases, and carbapenem-resistant *Acinetobacter baumannii*, *Pseudomonas aeruginosa*, and *Klebsiella pneumoniae* cases separately). **Results:** Time trends of infection control interventions showed increased rates in isolation of patients with MDR pathogens (*p* <0.001) and consumption of hand disinfectant solutions (*p* =0.001). The last four years of the study an annual decrease of VAP rate by 35.12% (95% CI: −53.52 to −9.41; *p* =0.01) was recorded, which significantly correlated not only with reduced trauma and cardiothoracic surgery patients (IRR:2.49; 95% CI: 2.09–2.96; *p* <0.001), but also with increased isolation rate of patients with MDR pathogens (IRR: 0.52; 95% CI: 0.27–0.99; *p =* 0.048), and hand disinfectants use (IRR: 0.40; 95% CI: 0.18–0.89; *p* =0.024). **Conclusions:** Infection control interventions significantly contributed to the decrease of VAP rate. Constant infection control stewardship has a stable time-effect and guides evidence-based decisions.

## 1. Introduction

Hospital-acquired pneumonia is an infection of the pulmonary parenchyma caused by pathogens that are present in hospital settings with an incubation period of at least 2 days. Ventilator-associated pneumonia (VAP) develops in intensive care unit (ICU) patients who have been mechanically ventilated for at least 48h [1]. Patients receiving mechanical ventilation are at high risk for complications, disability and poor outcome, longer stays in the ICU and hospital, and increased healthcare costs [2].

The accurate estimation of the incidence of VAP remains a challenge mainly because of subjective interpretation of clinical findings as well as because the diagnosis is made based purely on clinical criteria or via invasive lower airway cultures [3]. The type of ICU and patients also may impact the incidence of VAP; i.e., trauma and cardiothoracic surgery patients are at exceedingly high risk for VAP [4]. The United States National Healthcare Safety Network surveillance for the year 2012 estimated that the incidence of VAP ranged from 0.0–4.4 per 1000 ventilator-days depending on hospital unit [5]. According to the 2012–2017 International Nosocomial Infection Control Consortium surveillance study conducted in 523 ICUs in 45 countries worldwide, the mean incidence of VAP was estimated at 14.1 per 1000 ventilator-days [6]. However, as high as 18.3 episodes of VAP per 1000 ventilator-days were reported in a prospective observational study conducted in 27 ICUs in nine European countries in 2017 [7].

The microbial agents of VAP vary considerably and information about the microbiology of VAP serves to guide optimal antibiotic therapy. Most cases of VAP are caused by bacterial pathogens that normally colonize the oropharynx and gut, or are transmitted through healthcare personnel from other patients or environmental surfaces [8]. Antibiotic-resistant pathogens such as *Pseudomonas* and *Acinetobacter* species and methicillin-resistant *Staphylococcus aureus* are much more common after prior antibiotic treatment or prolonged hospitalization or mechanical ventilation [4,8].

The association between healthcare-associated infections and infection control interventions was investigated recently using observational and interrupted time-series analyses [9,10]. Various preventive healthcare strategies have been particularly assessed for VAP using this method [11,12]. The aim of the current study was to evaluate the association between the incidence of VAP, the incidence of MDR bacteremia, and specific infection control interventions in an adult ICU in a tertiary-care hospital in Greece.

## 2. Materials and Methods

### 2.1. Setting

This is a hospital-based study conducted prospectively from January 2013 to December 2018 in a 20-bed adults’ ICU of a 300-bed private tertiary-care hospital in Athens, Greece.

### 2.2. Interventions

Interventions were implemented throughout the study period and evaluated every month. Interventions consisted of: (1) surveillance of carbapenem-resistant *Acinetobacter baumannii*, *Pseudomonas aeruginosa*, and *Klebsiella pneumoniae*; (2) implementation of a VAP bundle, which included head elevation to 30°–45°, daily sedation vacation, daily assessment of readiness to wean, peptic ulcer disease prophylaxis, deep venous thrombosis prophylaxis, endotracheal tube aspiration techniques to keep ventilator circuits clean and dry, mouth hygiene through chlorhexidine 2%, and hand washing before and after providing healthcare to patients; (3) promotion of hand hygiene for all ICU patients; and(4) multi-body-site colonization screening cultures (pharyngeal, axillary-rectal, nasal) and isolation of patients with MDR bacteria.

### 2.3. Outcomes

The following outcomes were measured every month: (1) VAP rate as incidence per 1000 ventilator-days; (2) use of hand disinfectant solutions as liter (L) per 1000 patient-days; and (3) incidence of bacteremia per 1000 patient-days (total cases, total carbapenem-resistant cases, total carbapenem-resistant *A. baumannii, P. aeruginosa,* *K. pneumoniae* cases, separately).

### 2.4. Data Collection

Data were collected prospectively and monthly during the study period: use of hand disinfectant solutions from Pharmacy Department; number of VAP cases from Nurse Clinical Infectious Diseases Department; number of bacteremia from Microbiology Department. During the study period no changes were made in medical/nurse hospital quality procedures and laboratory diagnostic procedures.

### 2.5. Diagnostic Criteria for VAP

VAP was diagnosed based on a modified combination of both the 2013 Center of Disease Control and Prevention and Johanson criteria. A patient would be diagnosed with VAP if chest X-ray showed new infiltrates and at least two of the following six criteria were present: (1) fever (temperature > 38 °C); (2) abnormal white blood cells (WBCs) count; whether leukocytosis (>12,000/μL) or leucopenia (<2000/μL); (3) altered respiratory secretions; (4) elevated C-reactive protein (CRP) > 3 mg/L; (5) positive blood or respiratory culture; and (6) change in ventilator setting, including the Fractioned Inspired Oxygen (FiO_2_) (>50%), mean airway pressure (>14 cmH_2_O), and high positive end-expiratory pressure (PEEP): values > 6 cmH_2_O [13].

### 2.6. Detection of Bacteremia and Microbial Resistance

Bacteremia was detected through Gram stains and blood cultures. The isolation, identification, and antibiotic susceptibility testing were completedusing the automated VITEK 2 system (Biomerieux, Marcy-l’Étoile, France) and the resistance of selected bacteria to specific antibiotics was set out by using CLSI breakpoints. The assayused to determine the susceptibility of bacteria was recorded (Kirby–Bauer test, MIC semi-automated testing, E-test).

### 2.7. Definitions

VAP rate per month was defined as the number of VAP episodes per 1000 ventilator-days. Isolation rate of patients with MDR bacteria was defined as percentage of isolated patients with MDR per admissions (% isolations/admissions). Bacteremia was defined as a laboratory-confirmed bloodstream infection, either primary (not related to an infection at another body site) or secondary (thought to be seeded from a site-specific infection at another body site) [14]. New episode of bacteremia, within a month period, was defined due to a different pathogen strain or due to the same pathogen strain but with different phenotype of resistance. The incidence of total bacteremia is the sum of total Gram-positive and Gram-negative bacteremia. The incidence of total carbapenem-resistant Gram-negative bacteremia is the sum of the incidence of carbapenem-resistant *A. baumannii*, *P. aeruginosa*, and *K. pneumoniae* bacteremia. The incidence of total resistant Gram-positive bacteremia is the sum of the incidence of methicillin-resistant *S. aureus* plus vancomycin-resistant *Enterococcus* bacteremia. Hand hygiene includes the use of scrub disinfectant solutions with chlorhexidine, alcohol 70% disinfectant solutions with chlorhexidine, and/or simple soap.

### 2.8. Statistical Analysis

Investigation of time trends in the intervention and outcome variables during study period was initially performed, constituting the dependent variables. Time since the beginning of the study (in months) was the independent variable in the regression models and was entered through appropriate restricted cubic splines. Fourier series terms of time (1st and 2nd order) were also entered in the models to capture potential seasonality effects. In all cases, standard errors (SE) and corresponding 95% confidence intervals (CI) were derived using the robust (sandwich) variance estimator to adjust potential violations of models’ assumptions. Estimated values for start and end of the study period and corresponding 95% CIs were estimated through a simplification of the models where spline time terms were replaced by a single linear time trend or two piecewise linear terms to capture average long-term trend. A linear regression model was used for disinfectants consumption. For cases where the outcome of interest was VAP or bacteremia rates, Poisson regression models were used with number of cases as dependent variable and appropriate number of ventilation-days or patient-days, respectively, used as an offset after logarithmic transformation. For cases in which the percentage over total number of hospitalizations was the outcome of interest (isolations), binomial regression models were used with number of cases as dependent variable and the appropriate number of hospitalizations as binomial denominator. Associations between outcomes and interventions were investigated by introducing appropriate independent variables into the models. The effects of the independent variables were initially tested separately for current (month 0) and lagged values (months –1, –2 and –3). If the effects were statistically significant *(p* < 0.05) or indicative (0.05 < *p* < 0.10) for more than one case (e.g., in month 0 and in month –1) and association direction was the same (e.g., positive for both), average value was used as independent variable. In cases where direction of the association was different (e.g., positive for month 0 and negative for month –1), results of the respective models are presented separately. All *p*-values reported throughout the manuscript have not been adjusted for multiple testing. Analyses were conducted using Stata version 14.2 (Stata Corp., College Station, TX, USA).

## 3. Results

**Figure 1:**
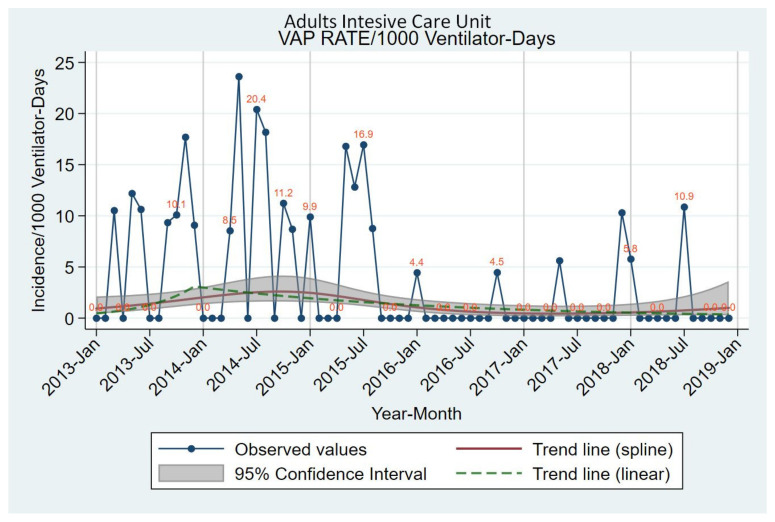
Observed values and estimated time trends of the incidence rate of VAP in the ICU, January 2013 to December 2018.VAP: ventilator-associated pneumonia; ICU: intensive care unit. Trends shown with dashed lines werederived from Poisson regression models with robust standard errors, seasonality terms and linear or piecewise linear long-term trend: log(N) = β_0_+β_1_t_−_+β_2_t_+_ +β_3_ × sin(2πt/12) + β_4_ × cos(2πt/12) + β_5_ × sin(4πt/12) + β_6_ × cos(4πt/12) + log(ventilator-days) with N being the number of cases and t being time since study start in months (t_−_ and t_+_ piecewise linear time terms). Trends shown with grey area werederived similarly but spline terms of time were used for long term trend instead of piecewise linear terms.

During the six-year study period, a total of 4754 admissions occurred in the ICU, of whom 63.23% underwent mechanical ventilation. Total number of VAP episodes was 31 among 3006 ventilated patients (1.03%) and the overall six-year VAP rate was 3.26 episodes of VAP/1000 ventilator days. Visualization of VAP incidence is displayed in Figure 1.

Distribution of admissions per type of ICU patients is shown in Table 1. Distribution of ventilated patients, mean ventilator-days, and VAP rate per year are shown in Table 2. The first set of results for each measure over time is summarized in Table 3 and Table 4. The relationship between VAP rates and concurrent or lagged (1–3 months) values of each process measure are summarized in Table 5 and Table 6.

From the annual number of admissionsin 2013, cardiovascular surgery and trauma patients accounted for 75.36% of ICU admissions and incidence VAP rate was estimated at 7.08 cases per 1000 ventilator-days (Table 1 and Table 2). In 2014 cardiovascular surgery patients decreased from 659 in 2013 to 411, while the number of trauma patients increased from 23 in 2013 to 131, which had an impact on the incidence of VAP, reaching 7.377 cases per 1000 ventilator-days. In the following years the annual incidence of VAP decreased from 5.331 in 2015 to 1.418 cases per 1000 ventilator-days in 2018 along with the percentage of cardiovascular surgery and trauma patients per total ICU admissions (from 44.10% in 2015 to 21.29% in 2018). The mean ventilator-days increased from 2.832 in 2013 to 4.596 in 2016 and decreased afterwards to 2.919 until 2018 (Table 2).

Table 3 shows the six-year time trends of the implemented interventions in the ICU. There was a statistically significant increase in the percentage of isolation of patients with MDR pathogens per 100 admissions(*p* < 0.001). Regarding hand hygiene, the use of scrub disinfectant solutions increased significantly (*p* = 0.001), while no significant difference in the use of alcohol disinfectant solutions (*p* = 0.286) was observed. Overall, the use of all hand disinfectant solutions in the ICU increased significantly (*p* = 0.001).

Table 4 shows six-year time trends of the incidence rate of VAP and bacteremia in the ICU. There was a significant decrease of the incidence of VAP rate by 35.12% per year (95% C.I.: −53.52% to −9.41%; *p* = 0.01). Overall, there was a significant increase in the incidence of total ICU bacteremia (*p* < 0.001), but no significant change in the incidence of total carbapenem-resistant Gram-negative pathogens and separately. From the relative change per year, there was a significant increase in the incidence of total carbapenem-resistant *A. baumannii* bacteremia only the last year of the study (*p* = 0.003), and of total carbapenem-resistant *K. pneumoniae* bacteremia the first two years of the study (*p*-value = 0.030). For resistant Gram-positive pathogens, the incidence was very low, almost zero for most time of the study, so a linear model could not be applied.

Table 5 shows the correlation between incidence of VAP, incidence of different bacteremia and type of ICU patients. For carbapenem-resistant *A. baumannii* bacteremia, every increase in their incidence per 1/1000 patient-days, current month, resulted in a statistically significant increase per 17% in the incidence of VAP rate (*p* = 0.045). For carbapenem-resistant *K. pneumoniae* bacteremia, every increase in their incidence per 1/1000 patient-days, three months earlier, resulted indicatively in 24% increase in VAP rate (*p*-value = 0.086). For total cardiothoracic surgery and trauma patients every increase per number of 10 resulted in 149% increase in VAP rate (*p* < 0.001). The same effect was observed for cardiothoracic surgery patients (*p* < 0.001) and trauma patients separately (*p* = 0.002).

Table 6 shows the correlation of VAP rate with infection control interventions. For every increase per 10% of isolations of patients with MDR pathogens, two months earlier, resulted in a significant decrease per 48% in VAP rate (*p* =0.048). Regarding the use of hand disinfectant solutions, the correlation was also negative for current month. Every increase in the use of scrub disinfectant solutions and all hand disinfectant solutions per 10 L/1000 patient-days resulted in a statistically significant decrease in VAP rate per 15% (*p* =0.028) and 60% (*p* =0.024), respectively. However, there was no significant association with the use of alcohol disinfectant solutions (*p* =0.079).

## 4. Discussion

This is a six-year observational study aiming to assess the relationship between infection control interventions and the incidence of VAP in a 20-bed adults ICU in Greece. From 2013 to 2018 a VAP-bundle was implemented in the ICU continuously audited to promote prevention measures along with an infection control program to promote hand hygiene, contact precautions, and strict isolation guidelines for patient with MDR pathogens.In 2013 and 2014 up to 80% of ICU admissions were trauma and cardiothoracic surgery patients, with an annual VAP rate of 7.08 and 7.377 cases per 1000 ventilator-days, respectively. The incidence of VAP changed dramatically downwards the following years to 21.29% in 2018, with annual VAP rate dropping to 1.418 cases per 1000 ventilator-days. Published data indicate that these groups of ICU patients demonstrate high risk for VAP which may contribute to excess mortality, even though these patients are often otherwise healthy [4].

From a multicenter study in Western European cardiothoracic ICUs, VAP was the most frequent nosocomial infection occurred in 2.1% of patients for an overall estimated rate of 13.9 VAP cases per 1000 ventilator-days [15]. Most cases of VAP are caused by bacterial pathogens that normally colonize the oropharynx and gut [8]. In the majority of patients with the proper functioning immunity, this microbial virulence alone does not account for the virulence of a disease [16,17]. The largestpart of the virulence of an infectious or chronic non-infectious disease is the result of the overactive inflammation response of the immune system, which turns local transient inflammation into systemic or chronic inflammation [18,19]. Overnutrition might be the driving force for such inflammatory transition, and slightly undernutrition may be preferred in reducing hyperinflammation [20,21].

Apart from the underlying condition for which the patient requires ventilation, an additional risk factor for VAP is the increased mechanical ventilation time and prolonged length of hospital stay [22]. In our study the duration of a patient’s mechanical ventilation is not consistent with VAP rate, with the mean ventilator-days increasing until 2016 and decreasing afterwards until 2018. In particular, 2016 had the highest mean value for the duration of mechanical ventilation and the lowest VAP rate, indicating indirectly the impact of preventive measures and infection control interventions along with the change of ICU type of patients.

Early clinical practice guidelinesfor VAP were based primarily on experts’ opinion derived from clinical experience, but throughout the years, the infectious diseases societies joined the respiratory, thoracic, and intensive care medicine associations to release shared documents [23,24]. In addition, novel approaches have been investigated for the reduction of VAP in the hospital setting using interrupted time-series method [19,20], such aschlorhexidine gluconate bathing [25] and dual hand hygiene audit [26]. However, VAP remains a frequent hospital-acquired infection worldwide and the increasing prevalence of MDR bacteria further drives the need to explore advances in diagnostic and empiric treatment strategies [27,28]. National surveillance data in Greece show a high prevalence of carbapenem-resistant pathogens in acute-care hospitals in this country, constituting a significant public health problem [29]. Additionally, in Greece the estimated incidence of HAIs accounted for 4.3% of cases, and in Europe for 3.7% of cases, according to two European point prevalence surveys conducted in acute care hospitals in 2016 to 2017 [30].

From our study, the most significant interventions in the ICU were the increased isolation rate of patients with MDR pathogens and the increased consumption of all hand disinfectant solutions, indicating adherence to the infection stewardship program. Furthermore, the most statistically significant outcome was the annual significant decrease of 35.12% in the incidence of VAP. To the best of our knowledge, our study provides the first-time data about the correlation of VAP not only with different types of ICU patients but also with different bacteremia and overall infection control interventions in an ICU. Correlation results of the applied time-series analysis showed that the reduction of trauma and cardiovascular surgery patients admitted in the ICU, the isolations of patients with MDR pathogens, and hand hygiene adherence guidelines resulted in a continuous decrease in VAP rate for four consecutive years.

As far the type of bacteremia is concerned, for carbapenem-resistant *A. baumannii* bacteremia, every increase in their incidence the current month alsoresulted in a significant increase in VAP rate, indicating that contact precautions would be beneficial on top of isolation measures, particularly for this pathogen. Recent publication using time series analysis did not show any difference regarding incidence of methicillin-resistant *S. aureus* or vancomycin-resistant *Enterococcus* bacteremia after cessation of contact precautions [31,32]. In our ICU, the increase of the incidence of carbapenem-resistant Gram-negative pathogens was not significant while the incidence of resistant Gram-positive pathogens was almost zero for most time of the study. In order to further reduce the incidence of healthcare-associated infections and particularly the prevalence of carbapenem-resistant pathogens, it is important to guide evidence-based decisions and implement tailored infection control policies [29,30].Our study has several strengths. We applied time series analysis to investigate the potential correlation between the incidence of VAP, infection control interventions, and MDR bacteremia. A clear strength is the six-year time period and the prospective collection of data. A potential limitation is the mixed type of patients admitted in our ICU, including cardiothoracic surgery and trauma patients. Seeing that the issue of adjusting *p*-values for multiple testing is controversial, we chose to report unadjusted *p*-values, thus some inflation of the Type I error beyond the typical 0.05 level cannot be excluded [33].

## 5. Conclusions

During a six-year study period we investigated the effect of infection control interventions and outcomes in VAP rate using time series data. Primarily, the reduction of trauma and cardiovascular surgery patients admitted in the ICU and secondarily the isolations and hand hygiene adherence guidelines resulted in a continuous decrease in VAP rate for four consecutive years. Favorable outcomes of infection control interventions implemented constantly in the ICU, such asthe increased isolation rate of patients with MDR pathogens and the increased consumption of all hand disinfectant solutions, harbored breakthrough infections from resistant pathogens. Further contact precaution measures could be applied for Gram-negative resistant pathogens in order to ameliorate the robust and constant effect of the infection stewardship program. Time series analysis is an important and safe tool to measure and evaluate data and interventions over time and to guide evidence-based decisions and infection control policies.

## Figures and Tables

**Table 1:** Annual number of admissions, trauma patients, cardiovascular surgery patients, and relevant percentages in ICU, January 2013 to December 2018.

	Admissions (n)	Trauma Patients (n)	CTS Patients (n)	%CTS Patients/Admissions	%(Trauma + CTS Patients)/Admissions
**2013**	905	23	659	72.82	75.36
**2014**	671	131	411	61.25	80.77
**2015**	737	12	313	42.47	44.10
**2016**	789	7	322	40.81	41.70
**2017**	783	9	215	27.46	28.61
**2018**	869	11	174	20.02	21.29
**6-year**	4754	193	2094	44.05	48.11

ICU: intensive care unit; CTS: cardiothoracicsurgery.

**Table 2:** Annual number of admissions, ventilated patients, mean ventilator-days, and VAP rates in ICU, January 2013 to December 2018.

	Admissions (n)	VP (n)	% VP/Admissions	Mean VD	VAP Rate/1000 VD
**2013**	905	688	76.02	2.83	7.08
**2014**	671	566	84.35	2.89	7.38
**2015**	737	352	47.76	3.73	5.33
**2016**	789	403	51.08	4.60	0.88
**2017**	783	514	65.64	4.19	1.39
**2018**	869	483	55.58	2.92	1.42
**6-year**	4754	3006	63.23	3.53	3.27

ICU: intensive care unit; VAP: ventilator-associated pneumonia; VP: ventilated patients; VD: ventilator-days.

**Table 3:** Time trend of interventions over time in the ICU, January 2013 to December 2018.

Time Trend					
ICU Interventions	EVSP January 2013 (95% CI)	EVEP December 2018 (95% CI)	*p*-Value	% Relative Change/Year (95% CI)	*p*-Value
**1.Isolations per 100 hospital admissions**					
% isolations	20.4(19.2 to 21.8)	27.6 (24.1 to 31.4)	<0.001	14.35 (10.62 to 18.22)	<0.001
				up to 6/2017	
				−12.34 (−24.77 to 2.13) after 6/2017	0.091
**2.Hand disinfectant solutions use (L/1000 patient-days)**					
Alcohol disinfectant sol	98.0	83.2	0.286	−2.50	0.286
	(80.9 to 115.1)	(68.5 to 97.9)		(−7.15 to 2.14)	
Scrub disinfectant sol	1.9	35.9	0.001	24.37 (18.64 to 30.10)	<0.001
	(7.4 to 11.3)	(17.7 to 54.2)		up to 8/2016	
				−22.85 (−34.36 to −11.35) after 8/2016	<0.001
All hand disinfectant sol	117.0	179.3	0.001	10.53	0.001
	(97.3 to 136.7)	(157.8 to 200.9)		(4.56 to 16.50)	

ICU: intensive care unit; sol: solution; EVSP: estimated value start period; EVEP: estimated value end period; CI: confidence interval1; sol: solution; L: liter; (1) All estimates were derived from binomial logistic regression models with robust standard errors, seasonality terms and piecewise linear long term trend: logit(π) = β_0_+β_1_t_−_+β_2_t_+_ +β_3_ × sin(2πt/12) + β_4_ × cos(2πt/12) + β_5_ × sin(4πt/12) + β_6_ × cos(4πt/12) with π being theprobability of isolation and t being time since study start in months (t_−_and t_+_ piecewise linear time terms). %Relative changes/year derived as [exp(12 × β_1_,_2_)–1] × 100%; (2) All estimates were derived from linear regression models with robust standard errors, seasonality terms, and piecewise linear long term trend: E[Y] = β_0_+β_1_t_−_+β_2_t_+_ +β_3_ × sin(2πt/12) + β_4_ × cos(2πt/12) + β_5_ × sin(4πt/12) + β_6_ × cos(4πt/12) with E[Y] being theexpected consumption value and t being time since study start in months (t_−_ and t_+_ piecewise linear time terms). Absolute changes/year was derived as β_1,2_ × 12.

**Table 4:** Time trend of the incidence rate of VAP and bacteremia in the ICU, January 2013 to December 2018.

Time Trend					
Outcomes	EVSP January 2013 (95% CI)	EVEP December 2018 (95% CI)	*p*-Value	% Relative Change/Year (95% CI)	*p*-Value
**1. VAP rate**					
Incidence of VAP/1000 ventilator-days	0.4 (0.1 to 2.0)	0.4 (0.1 to 1.3)	0.775	721.72 (18.45 to 5600.63) up to 12/2013	0.090
				−35.12 (−53.52 to −9.41) after 12/2013	0.011
**2.Incidence of bacteremia/1000 patient-days**					
Total bacteremia	18.2	32.8	<0.001	−3.81 (−15.16 to 9.07)	0.545
	(13.9 to 23.7)	(27.5 to 39.2)		up to 02/2016	
				28.57 (14.91 to 43.85) after 02/2016	<0.001
Total MDR bacteremia	1.9	2.3	0.678	34.51 (3.32 to 75.12)	0.028
	(1.2 to 3.0)	(1.2 to 4.2)		up to 10/2015	
				−18.86 (−38.09 to 6.34) after 10/2015	0.130
Total CR Gram (-) bacteremia	2.5	3.3	0.392	4.91 (−5.99 to 17.07)	0.392
	(1.7 to 3.5)	(2.1 to 5.1)			
Total MDR Gram (+) bacteremia	N/A	N/A	N/A	N/A	N/A
Total CR-Acbacteremia	0.8	1.8	0.256	−28.93 (−51.89 to 5.00) up to 01/2017	0.086
	(0.4 to 2.0)	(0.6 to 5.6)		209.33 (46.70 to 552.27) after 01/2018	0.003
Total CR-KlPn bacteremia	0.3	0.5	0.635	137.77 (8.59 to 420.63) up to 01/2015	0.030
	(0.1 to 1.2)	(0.1 to 1.7)		−28.44 (−51.84 to 6.33) after 01/2015	0.098
Total CR-PsA bacteremia	0.9	0.9	0.909	1.25 (−18.13 to 25.21)	0.909
	(0.4 to 1.8)	(0.4 to 2.1)			

ICU: intensive care unit; VAP: ventilator-associated pneumonia; MDR: Multidrug-resistant; CR-Ac: carbapenem-resistant *Acinetobacter baumannii*; CR-KlPn: carbapenem-resistant *Klebsiella pneumoniae*; CR-PsA: carbapenem-resistant *Pseudomonas aeruginosa;* N/A: not applicable; EVSP: estimated value start period; EVEP: estimated value end period; CI: confidence interval. All estimates were derived from Poisson regression models with robust standard errors, seasonality terms, and linear or piecewise linear long-term trend: log(N) = β_0_+β_1_t_−_+β_2_t_+_ +β_3_ × sin(2πt/12) + β_4_ × cos(2πt/12) + β_5_ × sin(4πt/12) + β_6_ × cos(4πt/12) + log(patient-days or ventilator-days) with N being the number of cases and t being time since study start in months (t_−_ and t _+_ piecewise linear time terms; when piecewise linear long-term trend was not required a single time term was used). % Relative changes/year were derived as [exp(12 × β_1_,_2_)−1] × 100%.

**Table 5:** Correlation between incidence of VAP, bacteremia from MDR pathogens, and type of ICU patients in an ICU, January 2013 to December 2018.

VAP Rate: Correlation with Bacteremia and Type of ICU Patients								
ICU	Per (n) Unit	Month 0	Month -1	Month -2	Month -3	IRR	95% C.I.	*p*-Value
**Incidence of bacteremia/1000 patient-days**								
Total bacteremia								n.s.
Total resistant Gram (+) &(−)								n.s.
Total CR Gram (-)								n.s.
Total CR-Ac	1	◊				1.17	(1.00, 1.36)	0.045
Total CR-KlPn	1				◊	1.24	(0.97, 1.57)	0.086
Total CR-PsA								n.s.
Number of ICU patients								
Cardiothoracic surgeries	10	◊				3.46	(2.71, 4.42)	<0.001
Trauma patients	10	◊				2.31	(1.36, 3.94)	0.002
Cardiothoracic surgeries + Trauma patients	10	◊				2.49	(2.09, 2.96)	<0.001

VAP: ventilator-associated pneumonia; IRR: Incidence rate ratio; CI: Confidence Interval; ICU: intensive care unit; CR-Ac: carbapenem-resistant *A.baumannii*; CR-KlPn: carbapenem-resistant *K. pneumoniae*; CR-PsA: carbapenem-resistant *P. aeruginosa*; ns: not-significant.Symbol ◊ denotes whether the association refers to the current month (month 0) value (1) incidence of bacteremia and (2) number of ICU patients, lagged values (months–1, –2, –3) or averaged values over more than one month. Incidence Rate Ratio (IRR) refer to increases in (1) incidence of bacteremia and (2) number of ICU patients, denoted in column labeled “per (n) unit”.All estimates were derived from Poisson regression models with robust standard errors, seasonality effects, and spline terms of time: log(N) = β_0_+β_1_V + β_2_S_1_(t) +β_3_S_2_(t) +β_4_S_3_(t) + β_5_ × sin(2πt/12) + β_6_ × cos(2πt/12) + β_7_ × sin(4πt/12) + β_8_ × cos(4πt/12) +log(ventilator-days)with N being the number of cases, t being time since study start in months, S(t) being spline terms of t, and V referring to the current month covariate (month 0) value, lagged values (months –1, –2, –3), or averaged values over more than one month. Incidence Rate Ratios (IRR) were derived as exp(n × β_1_) with n given in column labeled “per (n)”.

**Table 6:** Correlation of Incidence of VAP with infection control interventions in the ICU, January 2013 to December 2018.

VAP: Correlation with Infection Control Interventions								
Infection Control Interventions	Per (n) Unit	Month 0	Month -1	Month -2	Month -3	IRR	95% CI	*p*-Value
% isolations/admissions	10			◊		0.52	(0.27, 0.99)	0.048
L of alcohol disinfectant sol/1000 patient-days	10	◊				0.92	(0.83, 1.01)	0.079
L of scrub disinfectant sol/1000 patient-days	10	◊				0.85	(0.74, 0.98)	0.028
L of all hand disinfectant sol/1000 patient-days	10	◊				0.40	(0.18, 0.89)	0.024

VAP: ventilator-associated pneumonia; OR: odds ratio; CI: confidence interval; ICU: intensive care unit; L: liter. Symbol ◊ denotes whether the association refers to the current month (month 0) value, lagged values (months –1, –2, –3), or averaged values over more than one month. All estimates were derived from Poisson regression models with robust standard errors, seasonality effects, and spline terms of time: log(N) = β_0_+β_1_V + β_2_S_1_(t) +β_3_S_2_(t) +β_4_S_3_(t) + β_5_ × sin(2πt/12) + β_6_ × cos(2πt/12) + β_7_ × sin(4πt/12) + β_8_ × cos(4πt/12) +log(ventilator-days) with N being the number of cases, t being time since study start in months, S(t) being spline terms of t, and V referring to the current month covariate (month 0) value, lagged values (months –1, –2, –3) or averaged values over more than one month. Incidence Rate Ratios (IRR) were derived as exp(n × β_1_) with n given in column labeled “per (n)”.

## Data Availability

The data that support the findings of this study are available on request from the corresponding author, A.T. The data are not publicly available as data disclosure requires permission and ethical approval from Medical Ethical Committee of Athens Medical Center, Athens, Greece.
